# Mean Diffusivity of the Left Caudal Anterior Cingulate Cortex and Past Major Depressive Disorder in Adolescents: Evidence from the ABCD Study

**DOI:** 10.31586/jcn.2025.6206

**Published:** 2025-10-28

**Authors:** Shervin Assari, Alexandra Donovan, Babak Najand, Golnoush Akhlaghipour, Mario F Mendez

**Affiliations:** 1 Department of Psychiatry, Charles R Drew University of Medicine and Science, Los Angeles, CA, USA; 2 Department of Internal Medicine, Charles R Drew University of Medicine and Science, Los Angeles, CA, USA; 3 Department of Public Health, Charles R Drew University of Medicine and Science, Los Angeles, CA, USA; 4 Marginalization-related Diminished Returns Center, Los Angeles, CA, USA; 5 Montefiore Einstein’s Adult Neurology Residency Program, Bronx, New York, USA; 6 Department of Neurology, University of California Los Angeles (UCLA), Los Angeles, CA, USA; 7 Department of Psychiatry and Biobehavioral Sciences, University of California Los Angeles (UCLA), Los Angeles, CA, USA

**Keywords:** Anterior Cingulate Cortex, Mean Diffusivity, Adolescence, Major Depressive Disorder, ABCD Study, Gray Matter–White Matter Contrast, Diffusion MRI

## Abstract

**Background:**

Adolescence is a critical developmental stage for the emergence of major depressive disorder (MDD). Structural and diffusion neuroimaging studies have highlighted the anterior cingulate cortex (ACC) as a key region implicated in emotion regulation, stress reactivity, and mood processing. However, few studies have examined whether microstructural characteristics of the ACC, reflected by mean diffusivity (MD) within gray matter–white matter (GM–WM) contrast regions, are associated with depression in early adolescence.

**Objective:**

To examine whether mean diffusivity (MD) within the GM–WM contrast of the left caudal anterior cingulate cortex (ACC) is associated with a past diagnosis of MDD among adolescents in the Adolescent Brain Cognitive Development (ABCD) Study, after accounting for demographic, socioeconomic, and adversity-related factors.

**Methods:**

Data were drawn from adolescents with diffusion MRI–derived mean diffusivity measures and diagnostics. The independent variable was mean diffusivity (MD) of the GM–WM contrast in the left caudal ACC. The primary outcome was past MDD diagnosis based on structured psychiatric assessments. Covariates included age, sex, socioeconomic status (SES), and exposure to adverse childhood experiences (ACEs). Logistic regression models tested the association between ACC MD and past MDD. A secondary model evaluated the relationship between ACC MD and past suicide attempt.

**Results:**

Mean diffusivity of the left caudal ACC was associated with the odds of past MDD, independent of age, sex, SES, and adversity exposure. In contrast, ACC mean diffusivity was not associated with a history of suicide attempt.

**Conclusions:**

Increased mean diffusivity in the caudal ACC may indicate microstructural alterations associated with depressive vulnerability in adolescence. ACC tissue integrity may serve as a sensitive neural correlate of early-onset depression.

## 1. Introduction

Depression is among the most common and disabling psychiatric conditions to emerge during adolescence [1,2], a developmental period marked by profound neurobiological, psychological, and social change [3–5]. The onset of major depressive disorder (MDD) during this stage can disrupt emotional development, academic performance, and interpersonal relationships, often setting the stage for recurrent depressive episodes across the lifespan [6,7]. Identifying early neural markers of depression vulnerability is therefore essential for prevention and timely intervention, particularly before symptoms become chronic or severe [8].

Adolescent suicide, although a distinct phenomenon, shares several pathways with depression and remains one of the leading causes of death in youth [9]. The prevalence of suicidal thoughts and behaviors has risen markedly in recent years, reflecting a growing public health concern [10]. While suicide has its own social, cognitive, and neurobiological risk factors—such as impulsivity, hopelessness, trauma exposure, and access to lethal means [11,12]—depression remains one of its strongest predictors. Including suicide-related outcomes in studies of neural correlates of depression is therefore conceptually justified and clinically informative [13]. If certain neural features are associated with MDD but not suicide, or with depression and suicide, these distinctions may illuminate overlapping versus unique neurobiological and psychosocial pathways underlying these two highly interrelated yet partially distinct outcomes. Such findings would have important implications for identifying youth at differential risk and tailoring targeted prevention strategies.

Converging neuroimaging evidence points to the anterior cingulate cortex (ACC) as a central hub in the regulation of mood, emotion, and cognitive control [14]. The ACC integrates inputs from limbic and prefrontal systems to support emotion regulation, error detection, and self-monitoring—functions that are frequently altered in depression [15]. Structural and functional abnormalities in the ACC have been repeatedly reported in individuals with MDD, including atypical patterns of activity during emotional conflict and reduced structural integrity in both cortical layers and subcortical connections [16–18]. Emerging evidence suggests that the ACC may also play a role in suicidality, potentially through its involvement in emotional regulation, decision-making under distress, and sensitivity to social rejection or punishment [19]. The caudal portion of the ACC, located near the anterior midcingulate region, plays a particularly important role in conflict monitoring, behavioral control, and the regulation of negative affect [20]. Aberrant microstructure or function in this region has been linked to dysphoric mood, stress sensitivity, and altered executive control in both adult and adolescent populations [21].

Diffusion-based MRI metrics [22], such as mean diffusivity (MD) [23], provide insights into the microstructural organization of brain tissue that complement traditional morphometric measures like cortical thickness or volume. Elevated MD within cortical regions typically reflects reduced tissue integrity, altered myelination, or disrupted cellular organization. When assessed across the gray matter–white matter (GM–WM) contrast, MD captures the structural transition between cortical and subcortical compartments, offering a sensitive index of microstructural maturation. This approach can reveal subtle neurodevelopmental differences in areas related to emotional regulation and depression vulnerability that may not be evident through conventional structural measures alone.

### Aim

This study aimed to test whether MD within the gray matter–white matter contrast [24] of the left caudal ACC is associated with past MDD among adolescents participating in the Adolescent Brain Cognitive Development (ABCD) Study [25–34]. We hypothesized that MD, indicating microstructural integrity, would be associated with the odds of past MDD, independent of age, sex, socioeconomic status (SES), and exposure to adverse childhood experiences (ACEs). A secondary analysis examined whether ACC MD was also associated with past suicide attempt, to explore whether ACC microstructural alterations are specific to depression or shared across depressive and suicidal outcomes.

## 2. Methods

### Study Design and Sample

This cross-sectional study used baseline data from the Adolescent Brain Cognitive Development (ABCD) Study [25–34], a large, multisite investigation of brain, cognitive, and behavioral development in U.S. youth. Participants were recruited at ages 9 to 10 years from 21 research sites to approximate the sociodemographic diversity of the U.S. population. For the present analysis, we included adolescents with complete diffusion MRI data, valid diagnostic information on past MDD [zero or 1], and full data on covariates. Participants were excluded if they failed ABCD’s imaging quality-control (QC) procedures or if they had incomplete demographic, socioeconomic, or adversity information [35]. After applying these inclusion and exclusion criteria, the final analytic sample consisted of 7,448 adolescents with high-quality diffusion measures and diagnostic data suitable for multivariable modeling.

### Measures

#### Neuroimaging Variable

The primary neuroimaging variable was MD [36,37] within the gray matter–white matter (GM–WM) contrast of the left caudal ACC, derived from ABCD’s diffusion MRI data processed with the Destrieux cortical parcellation atlas [38] (aparc.a2009s). The corresponding variable name in the ABCD dataset is dmri_gwmdmd_lh_caudalanteriorcingulate in the data version 4.0. Mean diffusivity reflects the average rate of water molecule diffusion within tissue and serves as a marker of microstructural integrity. Higher MD typically indicates lower tissue coherence, reduced myelination, or increased extracellular space. When computed within GM–WM contrast regions such as the ACC, MD captures the transition zone between cortical and subcortical compartments, reflecting properties of both gray and white matter architecture. This measure provides a microstructural complement to traditional morphometric indices such as cortical thickness or volume.

#### Primary Outcome: Past Major Depressive Disorder

The primary outcome was a binary indicator of past MDD. MDD diagnosis was determined using the *Kiddie Schedule for Affective Disorders and Schizophrenia for DSM-5* (KSADS-5), a structured interview administered to both parents and adolescents [39,40]. Participants were coded as positive for past MDD if either informant reported a prior episode meeting diagnostic criteria for major depressive disorder during the baseline interview (ages 9–10). This approach ensures comprehensive capture of clinically meaningful depressive histories during early adolescence.

#### Secondary Outcome: Past Suicide Attempt

A secondary outcome was history of suicide attempt, derived from KSADS-5 items assessing self- or parent-reported suicidal behaviors as of ages 9–10 [39,40]. Responses were coded dichotomously to indicate the presence or absence of a lifetime suicide attempt [41]. This variable was analyzed separately to evaluate whether the observed ACC microstructural patterns were specific to depressive history rather than suicidality.

#### Covariates

All models were adjusted for key demographic and socioeconomic characteristics, including age (in months), sex (male or female), and socioeconomic status (SES). SES was operationalized using parental education (years of schooling) and both neighborhood- and household-level (HH) income categories. Initiation of puberty was assessed using the Pubertal Development Scale (PDS), harmonized with ABCD-derived [42–50] Tanner stage groupings [51–53]. Any indication of pubertal onset, based on the lowest threshold for PDS scores above which puberty is considered to have begun, was coded as “puberty initiated.” Because early adversity can influence both brain development and depression risk, models additionally controlled for the number of adverse childhood experiences (ACEs), which captured exposure to domains such as violence, neglect, and family instability [50,54]. Perceived discrimination was measured using a 7-item scale, with higher scores indicating greater perceived ethnic discrimination both within and outside of school settings [55–60]. Parent-reported financial difficulty, reflecting challenges paying for utilities, rent, or food, was scored on a 0–7 scale, with higher values indicating greater financial strain [61–66].

#### MRI Acquisition and Processing

MRI data were collected on 3-Tesla scanners from Siemens, GE, or Philips platforms, harmonized across ABCD sites using standardized acquisition parameters [67]. Diffusion-weighted images were acquired with multiple diffusion directions and b-values to capture both isotropic and anisotropic water diffusion properties. Image processing followed the ABCD Study’s standardized diffusion MRI (dMRI) pipeline [36,68]. The diffusion data underwent motion correction, eddy-current correction, and gradient nonlinearity adjustment. Diffusion tensors were fitted at each voxel to derive voxelwise maps of mean diffusivity (MD).

Surface-based analyses and parcellation were performed using *FreeSurfer* software (version 6.0) [69–71], applying the Destrieux atlas to define cortical regions of interest (ROIs) [38]. For each participant, the MD value within the GM–WM contrast of the left caudal ACC was extracted. Automated and manual quality-control checks were conducted following ABCD’s imaging quality control (QC) protocol, and participants with flagged or incomplete imaging data were excluded. This harmonized pipeline minimizes site-related variability and ensures consistency across participants and scanners.

#### Statistical Analysis

All statistical analyses were conducted using *R* (version 4.3.1) or *Stata* (version 18). Descriptive statistics were computed for all variables, stratified by past MDD status, and group differences were assessed using t-tests or chi-square tests, as appropriate. The primary analytic model used binary logistic regression to estimate the association between mean diffusivity in the left caudal ACC and past MDD, controlling for age, sex, SES, and ACEs. Mean diffusivity values were standardized (z-scored) to facilitate interpretation and comparability of effect sizes. Odds ratios (ORs) and 95% confidence intervals (CIs) were reported. A secondary model examined whether mean diffusivity of the same region was associated with a history of suicide attempt, using the same covariates. This tested whether observed associations were specific to depression rather than suicidality. Sensitivity analyses were performed in the absence of confounders. As the results did not change drastically, only the results of main analysis are shown. All statistical tests were two-tailed, with p < 0.05 considered statistically significant.

#### Ethics Statement

This study used publicly available, deidentified data from the Adolescent Brain Cognitive Development (ABCD) Study, a multisite longitudinal study approved by the Institutional Review Boards (IRBs) of all participating institutions. All ABCD participants provided written assent, and their parents or legal guardians provided written informed consent prior to participation. The current secondary analysis involved only deidentified data and therefore did not constitute human subjects research as defined by federal regulations; however, it was reviewed and acknowledged as IRB exempt under protocol [1761826-1] Multilevel Mechanisms of Intersectional Differences in Youth Suicidal Thoughts and Behaviors. All procedures were conducted in accordance with the Declaration of Helsinki and complied with all relevant ethical standards and institutional guidelines for research involving human participants.

## 3. Results

### Descriptive Characteristics

Our sample consisted of 7448 participants, 49.6% of which were male and 50.4% were female. Approximately 72.4% of adolescents had initiated puberty, while 27.6% had not. The majority of participants (76.2%) lived in married or partnered families, whereas 23.8% lived in single or non-partnered households. Regarding adversity exposure, 34.9% of adolescents had experienced at least one form of trauma, and 65.1% reported no trauma exposure. In terms of clinical outcomes, 1.7% of participants had previous MDD, based on KSADS.

Table 1 summarizes descriptive statistics for the analytic sample of 7,448 adolescents included in the study. Participants had a mean age of 119.35 months (approximately 9.9 years; SD = 7.49 months), consistent with the baseline age range of the ABCD Study. The average MD value within the gray matter–white matter contrast of the left caudal ACC was −0.17 (SD = 0.054), reflecting normalized diffusion values derived from the ABCD diffusion MRI pipeline.

Indicators of socioeconomic context showed considerable variability. The mean neighborhood socioeconomic status (SES), represented by the median family income from the Area Deprivation Index, was $79,006 (SD = $35,795; range = $0–$250,001). Household family income averaged $7,500 (SD = $2,280) when scaled per $1,000, and the highest parental education averaged 16.82 years (SD = 2.64), corresponding roughly to a college degree.

Indicators of psychosocial stress and adversity were generally low but variable. The mean discrimination score was 1.18 (SD = 0.41) on a scale from 1 to 5, suggesting that most participants reported minimal exposure to discrimination during the first study year. The mean financial difficulty score was 0.41 (SD = 1.02; range = 0–7), reflecting generally low levels of economic strain within this cohort.

### Bivariate Correlations

Table 2 presents the Spearman correlations among the main study variables, including MD within the gray matter–white matter contrast of the left caudal ACC, demographic variables, socioeconomic indicators, adversity exposures, and clinical outcomes.

MD values showed several small but statistically significant associations. Higher MD was modestly correlated with lower age (ρ = −0.032, p = 0.006) and male sex (ρ = −0.025, p = 0.030), suggesting slightly lower microstructural integrity among younger and female participants. MD was also positively associated with neighborhood income (ρ = 0.075, p < 0.001) and household income (ρ = 0.083, p < 0.001), and to a lesser extent with parental education (ρ = 0.067, p < 0.001). These positive associations indicated that higher family socioeconomic status (SES) was related to lower mean diffusivity, reflecting potentially greater microstructural organization of the ACC. MD showed small, nonsignificant associations with pubertal initiation, trauma exposure, or discrimination.

As expected, the SES variables were highly intercorrelated. Household income was strongly associated with parental education (ρ = 0.597, p < 0.001) and marital or partnered family status (ρ = 0.470, p < 0.001), and inversely related to financial difficulty (ρ = −0.454, p < 0.001), discrimination (ρ = −0.192, p < 0.001), and trauma exposure (ρ = −0.124, p < 0.001). These patterns collectively confirm the coherence of the socioeconomic indicators and their links to contextual adversities.

Regarding clinical outcomes, past MDD was positively associated with pubertal initiation (ρ = 0.030, p = 0.035), trauma exposure (ρ = 0.040, p = 0.002), discrimination (ρ = 0.079, p < 0.001), and financial difficulty (ρ = 0.057, p < 0.001), and negatively associated with household income (ρ = −0.047, p < 0.001) and parental education (ρ = −0.032, p = 0.012). These associations suggest that adolescents with a history of MDD were more likely to report adversity and lower socioeconomic advantage. The correlation between MD and past MDD was positive but small (ρ = 0.009, p = 0.509) and did not reach statistical significance in the bivariate model.

For suicide attempt, correlations showed a broadly similar but stronger pattern. Past suicide attempt was positively correlated with pubertal initiation (ρ = 0.041, p = 0.036), trauma (ρ = 0.047, p = 0.011), discrimination (ρ = 0.094, p < 0.001), and financial difficulty (ρ = 0.087, p < 0.001), and negatively correlated with household income (ρ = −0.107, p < 0.001) and parental education (ρ = −0.061, p = 0.001). Unlike depression, suicide attempt was not significantly related to MD (ρ = −0.008, p = 0.654).

### Multivariable Association with Past Major Depressive Disorder

Table 3 displays the results of the logistic regression model examining whether mean MD within the gray matter–white matter contrast of the left caudal ACC was associated with a history of MDD after adjustment for demographic, socioeconomic, and adversity-related factors.

After controlling for all covariates, ACC mean diffusivity was a significant positive predictor of past MDD (B = 5.050, SE = 1.693, p = 0.003). The odds ratio indicated that each unit increase in MD was associated with markedly higher odds of having a prior diagnosis of MDD (Exp[B] = 155.97, 95% CI = 5.65–4308.04). Although the confidence interval was wide, reflecting variability in diffusion values, the association remained statistically robust. This finding suggests that greater mean diffusivity—interpreted as lower microstructural integrity—within the caudal ACC is linked to elevated risk of a history of depression.

Among the demographic covariates, age was also significantly related to MDD (B = 0.038, p = 0.037), indicating that slightly older participants were more likely to report a history of depression. Sex, pubertal initiation, and neighborhood socioeconomic status were not significant predictors (all ps > .12).

Regarding psychosocial adversities, perceived discrimination displayed significance (B = 0.649, SE = 0.199, p = 0.001), with higher discrimination scores associated with a nearly two-fold increase in the odds of past MDD (Exp[B] = 1.91, 95% CI = 1.30–2.82). Exposure to trauma showed a marginal trend toward significance (B = 0.484, p = 0.069), suggesting that adolescents who had experienced any trauma were somewhat more likely to have a history of depression. Financial difficulty was not a significant predictor in the adjusted model (p =0.435).

Socioeconomic indicators, including household income (B = −48.779, p = 0.568), parental education (B = −0.030, p = 0.641), and marital or partnered family status (B = −0.174, p = 0.607), were not independently associated with past MDD once other variables were incorporated into the model.

### Multivariable Association with Past Suicide Attempt

Table 4 presents the results of the multivariable logistic regression model predicting past suicide attempt from mean diffusivity (MD) within the gray matter–white matter contrast of the left caudal anterior cingulate cortex (ACC) while adjusting for demographic, socioeconomic, and adversity variables.

After accounting for covariates, ACC mean diffusivity was not significantly associated with a history of suicide attempt (B = −1.181, SE = 2.735, p = 0.666). The direction of the effect suggested a nonsignificant trend toward lower MD among adolescents with a prior attempt, but the wide confidence interval (Exp[B] = 0.307, 95% CI = 0.001–65.405) indicated substantial uncertainty and instability in this estimate.

None of the demographic variables—including age (B = −0.021, p = 0.359), sex (B = 0.291, p = 0.389), or pubertal initiation (B = 0.468, p = 0.308)—were significantly related to suicide attempt in the adjusted model. Similarly, socioeconomic indicators such as neighborhood SES (B = 0.000, p = 0.884), household income (B = −122.334, p = 0.214), and parental education (B = 0.070, p = 0.407) were not significant predictors. Adolescents from married or partnered families showed lower odds of past suicide attempt (B = −0.655, p = 0.101), although this effect did not reach significance.

Among the psychosocial adversity indicators, discrimination emerged as the only significant predictor. Higher perceived discrimination was associated with increased odds of having a past suicide attempt (B = 0.540, SE = 0.272, p = 0.047; Exp[B] = 1.72, 95% CI = 1.01–2.92). Exposure to trauma showed a marginal association (B = 0.629, p = 0.063; Exp[B] = 1.88, 95% CI = 0.97–3.64), suggesting a possible trend toward higher suicide risk among youth reporting any traumatic experience. Financial difficulty (B = 0.152, p = 0.210) was not a significant independent predictor after adjusting for other variables.

## 4. Discussion

In this large sample of adolescents from the ABCD Study [25–34], mean diffusivity (MD) within the gray–white matter contrast of the left caudal ACC was associated with the odds of past MDD, even after adjusting for age, sex, SES, and exposure to adversities. This relationship was not observed for a history of suicide attempt, suggesting that ACC microstructural alterations may be more closely related to depressive vulnerability than to suicidality. These findings extend prior work linking anterior cingulate abnormalities to mood disorders by demonstrating that subtle differences in diffusion-based microstructure, rather than gross morphometry, are associated with depressive history among early adolescents.

The ACC plays a central role in the integration of emotional and cognitive processes, supporting regulation of affect, attention to internal states, and monitoring of conflict or error [72–81]. The caudal portion of the ACC, located in the anterior midcingulate region, is particularly involved in the regulation of negative emotion, behavioral control, and stress reactivity—all of which are disrupted in depression. MD within this region indexes microstructural integrity and organization of cortical tissue, providing a sensitive measure of developmental alterations that may precede macroscopic changes such as cortical thinning or volume loss [82–85]. Elevated MD likely reflects reduced tissue coherence, altered myelination, or increased extracellular diffusion, each of which may indicate maturational lag or neuroinflammatory processes relevant to depression pathophysiology [86].

Our findings align with prior adult neuroimaging studies reporting reduced integrity and altered connectivity of the ACC in major depressive disorder [87–89]. However, by focusing on adolescents, this study highlights that such microstructural deviations can already be detected during the developmental stage when MDD often first emerges. The pattern observed here suggests that ACC microstructure may serve as a trait-like marker of vulnerability, reflecting enduring neurobiological characteristics associated with risk for depressive episodes rather than acute symptom expression.

Extensive evidence points to the ACC—particularly its ventral and subgenual regions—as key nodes in the neurobiology of depression [90]. The subgenual ACC, located beneath the genu of the corpus callosum, has shown consistent abnormalities in both functional and structural imaging studies of adult depression. Reductions in subgenual ACC gray matter volume and metabolism have been observed in individuals with major depressive and bipolar disorders, and these alterations often persist after symptom remission [91,92]. Although the subgenual ACC was not directly examined in this study, its well-documented involvement in affective regulation lends theoretical support to our finding that adjacent regions of the ACC—including the caudal subdivision—may exhibit related microstructural features associated with depressive vulnerability.

Compared with the subgenual ACC, the caudal ACC is positioned more dorsally and is closely connected with prefrontal and limbic circuits involved in executive control and emotional awareness [93,94]. Alterations in this region’s microstructure may therefore represent a convergence point where emotion and cognition interact, consistent with observed difficulties in cognitive–affective integration among depressed adolescents. Together, these findings support the broader notion that depression involves distributed alterations across the cingulate–prefrontal network rather than being confined to a single subregion.

Several biological and environmental mechanisms may contribute to the observed association between ACC microstructure and depression. First, chronic stress and early adversity—both established risk factors for MDD—can affect myelination, glial proliferation, and synaptic remodeling in cingulate and prefrontal regions. Prolonged activation of stress pathways and inflammatory signaling may lead to reduced microstructural integrity, captured as higher MD [86]. Second, neurodevelopmental factors specific to adolescence, including synaptic pruning and ongoing white matter maturation, may render the ACC particularly sensitive to stress or genetic vulnerabilities [95–97]. Third, socioeconomic disadvantage may exacerbate these processes by shaping exposure to environmental stressors, nutritional factors, or chronic psychosocial strain. The persistence of the ACC–MDD association after adjustment for SES and adversity suggests that while these factors contribute, they do not fully account for the observed relationship.

Interestingly, MD in the left caudal ACC was not associated with a history of suicide attempt after covariate adjustment. This divergence may indicate that while ACC microstructure contributes to vulnerability for depression, suicide risk in youth may involve distinct neural mechanisms. Previous studies have implicated other cortical and subcortical regions—such as the ventromedial prefrontal cortex, insula, and amygdala—in the pathophysiology of suicidality, often through dysregulation of impulsivity, threat sensitivity, or decision-making under emotional stress [98,99]. It is possible that suicide risk reflects the interplay of depressive symptoms with behavioral disinhibition or environmental triggers, whereas ACC alterations primarily reflect mood dysregulation and cognitive control deficits characteristic of depression itself.

### Strengths and Limitations

A key strength of this study lies in its use of the ABCD Study, which provides a large, demographically diverse sample with harmonized neuroimaging and diagnostic data. The inclusion of multiple covariates—including demographics, SES, and adversity—reduces confounding and increases confidence that the observed associations are not merely artifacts of social or demographic differences. The use of mean diffusivity within GM–WM contrast represents a methodologically innovative approach that captures subtle microstructural features beyond those detectable through conventional volumetric or thickness analyses.

However, several limitations warrant consideration. The cross-sectional design precludes inference regarding temporal directionality or causation; it remains unclear whether ACC alterations precede, accompany, or result from depressive episodes, or at what age these depressive episodes occurred. The subgenual ACC, a region repeatedly implicated in mood disorders, was not available in the present parcellation, limiting subregional specificity. The reliance on self- or parent-reported diagnostic interviews may introduce recall or reporting biases, and the conversion of some measures from continuous to categorical may obscure more subtle findings. The study did not control for current depression, which may have added to the variability within results. Mean diffusivity is also a nonspecific marker, influenced by multiple tissue properties, and future research should incorporate complementary diffusion metrics such as fractional anisotropy or neurite density measures to improve biological interpretation. Finally, the analysis did not include functional connectivity data, which could elucidate whether altered ACC microstructure translates into disrupted network dynamics underlying emotion regulation.

### Implications and Future Directions

These findings underscore the potential of diffusion-based MRI metrics to identify early neural correlates of depression in adolescence. Elevated mean diffusivity within the ACC may reflect subtle neurodevelopmental deviations that confer vulnerability to future depressive episodes. Integrating diffusion data with longitudinal assessments will be essential for testing whether baseline ACC microstructure predicts subsequent onset, persistence, or recurrence of MDD. Additionally, multimodal approaches combining structural, diffusion, and resting-state functional MRI may clarify how microstructural abnormalities in the ACC influence functional connectivity within emotion-regulation networks.

From a translational standpoint, identifying early microstructural correlates of depression could inform prevention and intervention strategies. For instance, youth exhibiting elevated ACC MD and psychosocial risk factors might benefit from early psychosocial or cognitive-behavioral interventions targeting emotion regulation and stress resilience. Such research aligns with a growing focus on neurodevelopmentally informed prevention and precision mental health approaches that move beyond symptom-based classification.

## 5. Conclusion

In summary, mean diffusivity within the gray/white matter contrast region of the left caudal anterior cingulate cortex was associated with past major depressive disorder but not suicide attempt among adolescents in the ABCD Study. These findings suggest that microstructural alterations of the ACC may represent an early neurobiological correlate of depressive vulnerability during adolescence. Future longitudinal and multimodal studies are needed to determine whether such alterations precede the onset of depressive symptoms and to clarify their role in the developmental neurobiology of mood disorders.

## Figures and Tables

**Table 1.** Descriptive Data (n = 7448)

	Mean	Std. Deviation
MD-MRI	−0.17	.05
Age in months	119.34	7.49
Neighborhood SES	79006.72	35795.19
Discrimination Mean Yr1	1.18	0.41
Financial difficulty n	0.41	1.02
HH family income / 1000	0.007	0.002
Highest parental education (years)	16.82	2.64

**Note:** MD-MRI = Mean diffusivity within parcellation of gray matter-white matter contrast associated with cortical ROI left-caudal anterior cingulate; Neighborhood SES: Residential history derived - Area Deprivation Index: Median family income

**Table 2.** Bivariate Correlations (Spearman)

		MD-MRI	Age	Male	Pubertal initiation	Neighborhood income	Trauma	Discrimination	Financial Difficulty	HH Income	Parental Education	Married/Partnered Family	MDD
Age	rho	−.032**											
	p	0.006											
Male	rho	−.025*	.024*										
	p	0.030	0.030										
Pubertal Initiation	rho	−0.021	.133**	−.092**									
	p	0.103	0.000	0.000									
Neighborhood Income	rho	.075**	.050**	0.017	−.077**								
	p	0.000	0.000	0.136	0.000								
Trauma (Any)	rho	−0.005	0.013	−0.018	.052**	−.095**							
	p	0.671	0.261	0.122	0.000	0.000							
Discrimination	rho	−0.006	−.027*	.095**	.100**	−.161**	.045**						
	p	0.625	0.021	0.000	0.000	0.000	0.000						
Financial difficulty (n)	rho	−.038**	−0.005	0.010	.028*	−.298**	.157**	.108**					
	p	0.001	0.631	0.358	0.025	0.000	0.000	0.000					
HH Income	rho	.083**	.034**	0.003	−.064**	.589**	−.124**	−.192**	−.454**				
	p	0.000	0.004	0.778	0.000	0.000	0.000	0.000	0.000				
Highest parental education	rho	.067**	0.005	−0.002	−.057**	.478**	−.089**	−.144**	−.325**	.597**			
	p	0.000	0.672	0.863	0.000	0.000	0.000	0.000	0.000	0.000			
Married or partnered family	rho	0.011	−0.004	0.009	−.047**	.272**	−.125**	−.114**	−.256**	.470**	.268**		
	p	0.351	0.710	0.405	0.000	0.000	0.000	0.000	0.000	0.000	0.000		
Past MDD	rho	0.009	0.013	0.012	.030*	−.029*	.040**	.079**	.057**	−.047**	−.032*	−.033**	
	p	0.509	0.300	0.326	0.035	0.025	0.002	0.000	0.000	0.000	0.012	0.009	
Suicide Attempt	rho	−0.008	−0.015	0.014	.041*	−.070**	.047*	.094**	.087**	−.107**	−.061**	−.081**	.043*
	p	0.654	0.403	0.436	0.036	0.000	0.011	0.000	0.000	0.000	0.001	0.000	0.023

**Note:** MD-MRI = Mean diffusivity within parcellation of gray matter-white matter contrast associated with cortical ROI left-caudal anterior cingulate; Neighborhood SES: Residential history derived - Area Deprivation Index: Median family income; Past MDD: previous diagnosis of major depressive disorder.

**Table 3.** Logistic Regression with Past MDD as the Outcome

	B	S.E.	Sig.	Exp(B)	95% CI for	Exp(B)
MD - MRI	5.050	1.693	0.003	155.97	5.647	4308.043
Age months	0.038	0.018	0.037	1.039	1.002	1.077
Male	0.242	0.271	0.372	1.273	0.749	2.164
Pubertal Initiation	0.543	0.355	0.127	1.720	0.857	3.453
Neighborhood SES	0.000	0.000	0.958	1.000	1.000	1.000
DSM TRAUMA any	0.484	0.266	0.069	1.622	0.963	2.732
Discrimination Mean Yr1	0.649	0.199	0.001	1.914	1.297	2.824
Financial difficulty n	0.088	0.113	0.435	1.092	0.875	1.363
Income total family / 1000	−48.779	85.361	0.568	0.000	0.000	Unstable
Parent education years highest	−0.030	0.063	0.641	0.971	0.858	1.099
Married or partnered family	−0.174	0.337	0.607	0.841	0.434	1.628
Constant	−8.567	2.495	0.001	0.000		

**Note:** MD-MRI = Mean diffusivity within parcellation of gray matter-white matter contrast associated with cortical ROI left-caudal anterior cingulate; Neighborhood SES: Residential history derived - Area Deprivation Index: Median family income; Past MDD: previous diagnosis of major depressive disorder.

**Table 4.** Logistic Regression with Past Suicidal Attempt as the Outcome

	B	S.E.	Sig.	Exp(B)	95% CI for	Exp(B)
MD – MRI	−1.181	2.735	0.666	0.307	0.001	65.405
Age months	−0.021	0.023	0.359	0.979	0.937	1.024
Male	0.291	0.338	0.389	1.338	0.690	2.597
Pubertal Initiation	0.468	0.459	0.308	1.598	0.649	3.930
Neighborhood SES	0.000	0.000	0.884	1.000	1.000	1.000
DSM TRAUMA any	0.629	0.338	0.063	1.877	0.967	3.640
Discrimination Mean Yr1	0.540	0.272	0.047	1.716	1.007	2.923
Financial difficulty n	0.152	0.121	0.210	1.164	0.918	1.475
Income total family / 1000	−122.334	98.498	0.214	0.000	0.000	Unstable
Parent education years highest	0.070	0.085	0.407	1.073	0.908	1.267
Married or partnered family	−0.655	0.399	0.101	0.519	0.237	1.136
Constant	−3.209	3.050	0.293	0.040		

**Note:** MD-MRI = Mean diffusivity within parcellation of gray matter-white matter contrast associated with cortical ROI left-caudal anterior cingulate; Neighborhood SES: Residential history derived - Area Deprivation Index: Median family income
